# Bayesian Sample Size Calculations for External Validation Studies of Risk Prediction Models

**DOI:** 10.1002/sim.70389

**Published:** 2026-02-11

**Authors:** Mohsen Sadatsafavi, Paul Gustafson, Solmaz Setayeshgar, Laure Wynants, Richard D Riley

**Affiliations:** ^1^ Faculty of Pharmaceutical Sciences and Faculty of Medicine The University of British Columbia Vancouver British Columbia Canada; ^2^ Department of Statistics The University of British Columbia Vancouver British Columbia Canada; ^3^ British Columbia Centre For Disease Control Vancouver British Columbia Canada; ^4^ Department of Epidemiology, CAPHRI Care and Public Health Research Institute Maastricht University Maastricht the Netherlands; ^5^ Department of Development and Regeneration KU Leuven Leuven Belgium; ^6^ Department of Applied Health Sciences, School of Health Sciences, College of Medicine and Health University of Birmingham Birmingham UK; ^7^ National Institute for Health and Care Research (NIHR) Birmingham Biomedical Research Centre Birmingham UK

**Keywords:** Bayesian statistics, decision theory, risk prediction, sample size, uncertainty

## Abstract

Contemporary sample size calculations for external validation of risk prediction models require users to specify fixed values of assumed model performance metrics alongside target precision levels (e.g., 95% CI widths). However, due to the finite samples of previous studies, our knowledge of true model performance in the target population is uncertain, and so choosing fixed values represents an incomplete picture. As well, for net benefit (NB) as a measure of clinical utility, the relevance of conventional precision‐based inference is doubtful. In this work, we propose a general Bayesian framework for multi‐criteria sample size considerations for prediction models for binary outcomes. For statistical metrics of performance (e.g., discrimination and calibration), we propose sample size rules that target desired expected precision or desired assurance probability that the precision criteria will be satisfied. For NB, we propose rules based on Optimality Assurance (the probability that the planned study correctly identifies the optimal strategy) and Value of Information (VoI) analysis, which quantifies the expected gain in NB by learning about model performance from a validation study of a given size. We showcase these developments in a case study on the validation of a risk prediction model for deterioration among hospitalized COVID‐19 patients. Compared to conventional sample size calculation methods, a Bayesian approach requires explicit quantification of uncertainty around model performance, and thereby enables flexible sample size rules based on expected precision, assurance probabilities, and VoI. In our case study, calculations based on VoI for NB suggest considerably lower sample sizes are required than when focusing on the precision of calibration metrics. This approach is implemented in the accompanying software.

## 1 Introduction

Developing risk prediction models or validating existing models in a new population represents a significant investment in time, resources, and expertise. Like other empirical experiments, the design of such studies should be based on objective, transparent, and defensible principles. A particular aspect of study design is the sample size of such studies. The field has witnessed significant recent developments on this front [1, 2, 3, 4, 5, 6, 7, 8]. An example is the multi‐criteria approach by Riley et al. for the sample size required for external validation studies, targeting pre‐specified widths of the 95% confidence intervals (95% CI) around metrics of model performance [2, 3, 9]. For binary outcomes, such metrics are related to discrimination, calibration, and net benefit (NB). The sample size required for each component is computed separately, with the largest one used as the final requirement [3].

In addition to target precision criteria, each component of this approach requires as input an assumed value of the metric of interest in the anticipated sample. In reality, we do not know the true value of model performance metrics with certainty (otherwise, there would be no need to conduct a validation study in the first place). Further, due to sampling variability, the precision obtained in one particular validation study may differ substantially from the targeted precision for the calculated sample size. Thus, there is more uncertainty to be accounted for than the Riley criteria allow.

A Bayesian approach to sample size determination allows this uncertainty to be accounted for, which is advantageous on multiple fronts. First, it enables the full use of existing information on model performance at the time the study is designed, rather than forcing the investigator to express their knowledge as fixed known values. Second, it enables users to choose from different classes of sample size rules, namely those that target the expected values of precision targets, as well as “assurance”‐type rules for the probability of meeting (or exceeding) precision targets. The latter can be insightful as focusing on expected values alone does not guarantee that the desired precision will be achieved in one particular dataset, and the investigator may want a stronger assurance against this. Finally, when assessing clinical utility, the relevance of precision‐based criteria is challenged [10, 11]. A Bayesian approach enables the use of novel, decision‐theoretic approaches based on the Value of Information (VoI) analysis, which focuses on the expected gain in clinical utility from increasing sample size [12].

Classically, Bayesian approaches for power and sample size calculations were developed in the context of experimental studies aimed at interrogating a null hypothesis [13, 14, 15, 16]. Risk model validation studies, however, are not generally hypothesis‐driven; rather, the focus is on the precise estimation of a variety of metrics of model performance. Precision‐based Bayesian approaches for sample size calculations have been developed for diagnostic studies. Examples include Bayesian assurance methods targeting posterior interval width around metrics of test accuracy [17, 18], as well as for diagnostic studies in the absence of a gold standard where model identifiability is a challenge [19]. However, unlike diagnostic tests that typically deal with binary or categorical test values, risk prediction models generate continuous estimates of outcome risk. As such, Bayesian sample size considerations in this context are worth exploring.

Hence, in this article, we propose a Bayesian version of the sample size formula by Riley et al., which enables the investigator to (1) incorporate their uncertainty around the assumed model performance in calculations, (2) use sample size rules that target assurance probabilities for meeting chosen criteria, and (3) use rules based on the VoI analysis for clinical utility. In line with the contemporary practice in risk prediction development and validation, we adopt a mixed Bayesian‐likelihood (aka hybrid) perspective: We use existing evidence (prior) to quantify our uncertainty about model performance, but we assume that once the future sample is obtained, we will solely rely on it (the likelihood). We will discuss the implications of a fully Bayesian approach, where the likelihood and prior are combined. The proposed framework can be used in two general ways: To quantify the anticipated precision or VoI outcomes from a planned study if the sample size is fixed (as is the case with validation studies that are based on already collected data), or to determine the minimum sample size that achieves the pre‐specified precision or VoI criteria. This framework can also be used in a hybrid form: To determine the sample size based on certain criteria (e.g., targeting CI width for the c‐statistic and calibration slope), and to investigate the consequence of the chosen sample size using other criteria (e.g., VoI analysis for NB). Calculations can be done for the entire sample, as well as within subgroups, e.g., imposing fairness criteria around the target precision of estimates among minority sub‐groups.

The rest of this manuscript is structured as follows. First, we briefly review common metrics of model performance and the multi‐criteria sample size formula by Riley et al. [3]. We will then introduce our Bayesian extension of this framework based on various sample size rules. We propose a general approach for characterizing uncertainty around model performance based on commonly reported information from previous studies, and outline two Monte Carlo‐based sampling algorithms for drawing from the corresponding distributions. A case study based on a model for predicting COVID‐19 deterioration showcases the developments. We conclude by suggesting further areas for research.

## 2 Methods

In this section, we review the context, the common metrics of the model performance, Riley's sample size formulas for external validation studies, and our proposal for Bayesian sample size determination.

### 2.1 Context

We focus on the external validation of a risk prediction model for predicting the risk of a binary outcome. A risk prediction model is a deterministic mapping function returning an estimate of the conditional risk (henceforth referred to as “predicted risk”), denoted by π, of a binary outcome Y (0: No event, 1: Event) based on an individual's covariate (predictor) patterns. As is the standard in contemporary practice, predicted risks from the model are taken as fixed values.

An external validation study (henceforth referred to as the validation study for brevity) quantifies the performance of an existing model in a new sample. Given that such a study does not involve learning about the relationship between predictors and the outcome, the complexity of the prediction model is not of relevance. The prediction model may have been developed using regression, machine learning, or AI‐based methods. The performance of the model is typically summarized in terms of metrics related to discrimination, calibration, and clinical utility (NB). We are concerned with the relationship between the sample size of a validation study and the anticipated precision of metrics related to discrimination and calibration, as well as the expected gain in NB.

The validation sample $D_{N}$ of size N participants can be seen as N pairs of predicted risks and observed results: $D_{N}={\left\{(\pi_{i},Y_{i})\right\}}_{i=1}^{N}$. For ease of exposition, we assume one model is being evaluated. This framework can be extended to multi‐model comparisons with relative ease, but we leave this to subsequent exploration.

### 2.2 Common Metrics of Model Performance

Most model development and validation studies report the following metrics along with associated uncertainty:

1. *Outcome prevalence*: ϕ:=𝔼(Y).
2. *Calibration function* h(): This is the function that returns the conditional outcome risk given predicted risk: h(π)=P(Y=1|π). This function converts the predicted risk for the ith individual, $\pi_{i}$, to the “calibrated risk” for that person, which we denote by $p_{i}$. $p_{i}:=h(\pi_{i})$. For a given predicted risk π, the corresponding calibrated risk p is the average outcome risk across all individuals with that predicted risk (for a calibrated model $\forall i:p_{i}=\pi_{i}$).
  As needed, by h we refer to parameters describing h(). Commonly, including in Riley's sample size formula, h() is modeled linearly on the logit scale: logit(h(π))=α+βlogit(π), and thus h consists of an intercept (α) and slope (β). Sometimes studies report β and “mean calibration”, that is, 𝔼(Y−π), or β and observed‐to‐expected outcome ratio (O/E:=𝔼(Y)/𝔼(π)), but these are equivalent as for a fixed value of β, knowing any of calibration intercept, mean calibration, or O/E ratio, is enough to specify the calibration line. In other instances, h() is modeled more flexibly using non‐parametric methods (e.g., based on LOESS smoothing) [20]. Riley et al., while determining the sample size based on a logit‐linear calibration function, suggest visually inspecting the variability in the anticipated smoothed curves once the sample size is determined [2].
3. *c‐statistic* (c): This is a measure of the discriminatory performance of the model, and is the probability of concordance between the ranking of predicted risks and outcomes among a randomly chosen pair. Formally, $c:=P(\pi_{2}>\pi_{1}|Y_{2}=1,Y_{1}=0)$ with $\left(\pi_{1},Y_{1}\right)$ and $\left(\pi_{2},Y_{2}\right)$ being two randomly selected pairs of predicted risks and responses.
4. *Net benefit (NB)*: NB is a measure of clinical utility that is calculated for a given risk threshold for decision‐making. At a chosen risk threshold z, there are at least three treatment strategies concerning the use of the model: Treat no one (with a default NB of 0), use the model to treat individuals whose predicted risk is above the threshold (π≥z), or treat all. Vickers and Elkin argued that the choice of risk threshold for treatment implies an exchange rate between the utilities of a true and a false positive classifications, which enables the calculation of the NB of these strategies [21]. Details of the logic behind NB calculations are provided elsewhere [21], with a brief excerpt provided in the Appendix A. In a nutshell, after indexing the above‐mentioned three strategies by, respectively, 0, 1, and 2, the NB() function, retuning NB in true positive units, can be written as (for brevity of notation, in what follows, we drop the notation that would indicate NB‐related calculations are based on the chosen risk threshold):
$$\begin{array}{ll} & NB(k)\\ & \;=\left\{\begin{array}{ll} 0 & k=0\;\text{(treat no one)}\\ \phi se-\frac{z}{1-z}(1-\phi )(1-sp) & k=1\;(\text{use model to decide})\\ \phi -\frac{z}{1-z}(1-\phi ) & k=2\;(\text{treat all}) \end{array}\right., \end{array}$$
  where se:=P(π≥z|Y=1) and sp:=P(π<z|Y=0) are the sensitivity and specificity of the model at the chosen threshold, respectively.

An important difference between NB and other metrics of model performance is that NB is a decision‐theoretic measure. If using the model has a higher expected NB over other strategies (irrespective of uncertainties), then it is expected to confer clinical utility, and so the decision would be to use the model. On the contrary, metrics of model performance, such as c‐statistic or calibration function, do not consider the decision‐making context.

### 2.3 Riley's Sample Size Formulas

Citing the commonality of the above‐mentioned metrics in risk modeling studies, Riley et al. structured their multi‐criteria equations around desired precision levels (width of 95% CI) around these metrics [3]. In particular, the following equations were suggested for approximating the standard deviation of the sampling distribution of the estimators (i.e., standard error [SE]):

$$\text{SE}(c)=\sqrt{\frac{c(1-c)\left[1+(N/2-1)(\frac{1-c}{2-c})+\frac{(N/2-1)c}{1+c}\right]}{N^{2}\phi (1-\phi )}},$$

$$\text{SE}(\log(O/E))=\sqrt{\frac{1-\phi }{N\phi }},$$

and

$$\text{SE}(\beta )=\sqrt{\frac{I_{\alpha }}{N(I_{\alpha }I_{\beta }-I_{\alpha ,\beta }^{2})}},$$

where $I_{\alpha }=𝔼\left(p(1-p)\right)$, $I_{\beta }=𝔼\left(\text{logit}{\left(\pi \right)}^{2}p(1-p)\right)$, $I_{\alpha ,\beta }=𝔼\left(\text{logit}(\pi )p(1-p)\right)$, and p=h(π).

The SE equations are used to construct Wald‐type 95% CIs that are targets of sample size calculations (e.g., c±1.96.SE(c) as the bounds of the 95% CI for c‐statistic). For each component, the required sample size is the one that corresponds to a pre‐specified CI width. Assuming all measures are of interest, the final sample size is decided by the largest N among each component (and by checking whether the subsequent variability of calibration curves is acceptable—as explained above). Riley et al. also proposed targeting the desired CI width around NB. However, a Bayesian approach facilitates using decision‐theoretic sample size rules for NB, addressing the criticisms around the relevance of conventional inferential methods for measures of clinical utility [10, 11].

Note that the calculations require assumptions about the anticipated model performance (e.g., c‐statistic, calibration slope) and the distribution of predicted risks in the target population. The authors suggested using single point‐estimates for the true model performance values, and one chosen distribution for predicted risks. We now propose a Bayesian approach that enables modeling uncertainties around these quantities.

### 2.4 Going Bayesian

The main premise of a Bayesian approach is to consider explicitly our uncertainties around model performance in the target population. All sample size targets (e.g., 95% CI width around calibration slope) ultimately depend on the joint distribution of predicted risks and outcomes in the target population. Let $P_{\theta }(\pi ,Y)$ represent our knowledge about this joint distribution, indexed by parameters θ. In our Bayesian approach, θ is a random entity summarizing our current knowledge about this joint distribution (which encompasses our beliefs about model performance in the target population). We plan to learn about θ by collecting a sample of data $D_{N}$ from N participants in the target population. A Bayesian approach treats θ, $D_{N}$, and therefore any sample size target derived from $D_{N}$ (e.g., CI width of the c‐statistic, NB of the model) as random entities.

We refer to the anticipated distribution of sample size targets across future samples as “pre‐posterior” distributions (also known as prior predictive distributions). For example, the CI width around the calibration slope will be a random variable as it is derived from the randomly generated $D_{N}$. The pre‐posterior distribution of this quantity is its distribution across future $D_{N}$s. This distribution is a complex function of θ, and closed‐form derivations are generally unavailable. Instead, our Bayesian approach is operationalized via Monte Carlo simulations, which involves the following steps: We specify P(θ), our current (prior) information about model performance in the target population. Repeatedly, we sample from P(θ) and simulate a random validation sample from $P(D_{N}|\theta )$. From this, sample size targets (e.g., 95% CI widths) are quantified and recorded. Table 1 provides an algorithmic description of these steps.

**TABLE 1:** Bayesian Monte Carlo algorithm for drawing from the pre‐posterior distribution of precision targets.

1. Assign a distribution to prevalence, c‐statistic, and calibration function representing current knowledge (e.g., based on reported point estimates and confidence bands from previous development or validation studies). If the calibration function is specified as a line on the logit scale, this can be one of the following.
• Distributions for calibration intercept and slope.
• Distributions for O/E ratio and calibration slope^a^.
• Distributions for mean calibration and calibration slope^a^.
2. Assign a distribution type for calibrated risks p in the source population^b^.
3. Obtain a sample of size S for θ: $\theta^{\left(j\right)}=\{\phi^{\left(j\right)},c^{\left(j\right)},h^{\left(j\right)}\},j=1,2,\ldots ,S$. Optional: Use parametric bootstrapping to induce correlation among {ϕ,c,h}.
4. For j=1 to S (number of Monte Carlo simulations).
(a) Derive the parameters of $P^{\left(j\right)}(p)$, the distribution of calibrated risks in this iteration, given $\phi^{\left(j\right)}$ and $c^{\left(j\right)}$, given the distribution type assigned in step 2.
^a^ Sample‐based method
(b) Draw N observations for calibrated risks: $p_{i}^{\left(j\right)}∼P^{\left(j\right)}(p),i=1,2,\ldots ,N$. Draw corresponding response values $P(Y_{i}^{\left(j\right)})∼\text{Bernoulli}(p_{i}^{\left(j\right)})$. Calculate the corresponding values of π: $\pi_{i}^{\left(j\right)}={h^{\left(j\right)}}^{-1}(p_{i}^{\left(j\right)})$. Construct ${D_{N}}^{\left(j\right)}={\left\{(\pi_{i}^{\left(j\right)},Y_{i}^{\left(j\right)})\right\}}_{i=1}^{N}$ as the validation sample.
(c) Using ${D_{N}}^{\left(j\right)}$, construct and record precision targets (CI widths): $ciw^{\left(j\right)}=g({D_{N}}^{\left(j\right)})$ for each metric of interest.
^a^ Two‐step approach
(b) For any metric $\theta_{k}$, specify $P({\hat{\theta }}_{k}|\theta^{\left(j\right)})$ using method of moments, with the first moment being true value of $\theta_{k}$ from $\theta^{\left(j\right)}$, and the second moment being $SE^{2}(\theta_{k})$ (and a choice of distribution type^c^). Obtain ${\hat{\theta }}_{k}$ as a draw from this distribution.
(c) Plug ${\hat{\theta }}_{k}$ into the relevant SE equation to compute the precision criterion (CI width). Record this value.
Next j
5. Process the draws from the precision targets according to the sample size rule specified:
– For expected CI width criteria: $E_{CIW}=\sum_{j=1}^{S}ciw^{\left(j\right)}⁄S$.
– For quantile (assurance) CI width criteria: $Q_{CIW}={\hat{F}}_{ciw}^{-1}(q)$, where ${\hat{F}}_{ciw}$ is the empirical CDF of ciws and q is the desired quantile (e.g., 0.9).

^a^ For a given value of prevalence, both these specifications result in a value for 𝔼(π), thus requiring finding parameters of h() in $𝔼(\pi )=\int_{0}^{1}h^{-1}(p)f(p)dp$. For logit‐linear calibration function, h(π)=expit(α+βlogit(π)). In our implementation, solving for α given 𝔼(π) and β is programmed as univariate root‐finding.

^b^ Currently, Beta, Logit‐normal, and Probit‐normal distributions are modeled in the accompanying R package.

^c^ Central Limit Theorem justifies Normal distribution as the default. This is indeed compatible with the Wald method of constructing a CI.

The above steps result in draws from the pre‐posterior distributions of sample size targets. If the sample size is fixed, these distributions are processed according to various rules (e.g., the expected CI width around O/E ratio will be its average across all simulated $D_{N}$s). Sample size calculation is a stochastic inverse problem: Find the smallest N that satisfies a set of sample size rules.

#### 2.4.1 Uncertainty Characterization: Specifying P(θ)

The Bayesian approach requires specifying P(θ), the distribution of parameters that govern $P_{\theta }(\pi ,Y)$. This parameterization can be done in different ways. For example, if a (pilot) sample from the target population is available, one can adopt a non‐parametric approach based on the Bayesian bootstrap. In this scheme, θ is the vector of weights assigned to each observation in the pilot sample. These weights are random with a distribution of P(θ)∼Dirichlet(1,…,1). The empirical distribution of this weighted sample can be considered as a random draw from the distribution of the population. The validation sample $D_{N}$ can then be obtained from sampling with replacement. Details of such a two‐level resampling approach is provided elsewhere [22].

Our focus here is on parametric modeling based on summary statistics from previous studies. Noting that P(π,Y)=P(π)P(Y|π), with Y|π∼Bernoulli(h(π)), an intuitive way of parameterizing θ is via specifying P(π), the distribution of predicted risks, and P(h), the distribution of parameters defining the calibration function h(.). However, specifying the distribution of predicted risks in the target population is not straightforward, as this distribution is affected by model performance in complex ways. Fortunately, the specification of our knowledge in terms of outcome prevalence, c‐statistic, and calibration function (the same components used in Riley's equations), that is, defining θ={ϕ,c,h}, can, under mild regularity conditions, fully identify P(π,Y). The regularity conditions are as follows:

1. h(.) is monotonically ascending (under the assumption of logit‐linearity, this is satisfied as long as the calibration slope is positive), and
2. P(p), the distribution of calibrated risks, is quantile‐identifiable; that is, any two quantiles of the distribution are sufficient for uniquely identifying it. Typical distributions for risks, including Beta, Logit‐normal, and Probit‐normal, satisfy this requirement [23].

The first condition guarantees that the c‐statistic relating π to Y (which is often reported) is equal to the c‐statistic for P(p) (as the c‐statistic is invariant under monotonic transformation of predictor values). The second condition is a requirement for the identifiability of P(p) given its mean (prevalence) and c‐statistic [23].

As an example of this identifiability, consider an outcome prevalence of 0.25, c‐statistic of 0.75, calibration slope of 1.1, and O/E ratio of 0.9. Assuming predicted risks have a Logit‐normal distribution, and given our assumption on the logit‐linearity of h(), the calibrated risks will also have Logit‐normal distribution. The parameters of the latter are uniquely identifiable from {ϕ,c}, which resolves to P(p)∼Logitnorm(−1.3302,1.0395) (the *mcmapper* R package implements our proposed numerical algorithms for this mapping [24]). As well, there is a 1:1 mapping between the O/E ratio and calibration intercept. Given the calibration slope of 1.1 and the specified distribution for p, an O/E ratio of 0.9 uniquely maps to a calibration intercept of −0.089 (see footnote of Table 1 for additional information). Thus, to generate a random validation sample, one can sample N calibrated risks from P(p), generate corresponding response values as $Y_{i}∼\text{Bernoulli}(p_{i})$ and compute predicted risks as $\pi_{i}=h^{-1}(p_{i})$. $\left(\pi_{i},Y_{i}\right)$s created this way will be a realization of $D_{N}$.

Given this identifiability, characterizing our prior information involves specifying the joint distribution P(ϕ,c,h). Ideally, the previous analysis would report both the value and an estimated covariance matrix of $\hat{\theta }$; that is, there would be joint inference about the three elements of θ. These would then become the prior mean and covariance matrix of the joint prior for θ. In typical practice, however, joint inference on such parameters is not reported, but probability distributions for individual components can readily be constructed from existing information. For example, for outcome prevalence, if from a previous study with sample size of n, m individuals experience the outcome, our knowledge can be specified as Beta(m,n−m). For other components, point estimates and reported CI bounds can be used, along with an assumed distribution type, to construct distributions. Examples include specifying a Log‐normal distribution for O/E ratio, Normal distribution for calibration slope, and Beta distribution for c‐statistic based on reported point estimates and bounds of 95% CI. The accompanying software provides a flexible way of specifying such distributions, accepting specification of distribution parameters, moments, or the mean and upper bound of the 95% CI.

Once marginal distributions are constructed, a simple strategy would be to complete the prior specification by imposing a priori independence between the three components. An optional strategy, which would be more faithful to the true data‐generating mechanism, would involve a parametric bootstrap procedure to recover interdependence amongst the parameters. In this approach, one simulates multiple samples given $\theta =\hat{\theta }$, and for each sample records the ensuing estimates of $\hat{\theta }$. The empirical correlation matrix of these simulated estimates can then be taken as the prior correlation matrix, instead of simply presuming an uncorrelated prior. Supporting Information—Section 1 provides an algorithmic description of this approach.

This algorithm in itself quantifies the uncertainty for a population that is exchangeable with the population(s) from which evidence on model performance is collected. If the evidence is collected from a single population, this specification assumes model performance is the same between the source and target populations. On the other hand, if current evidence is synthesized from multiple populations using meta‐analytic techniques (as in our case study below), this specification assumes the target population is a random draw from the distribution of the meta‐population. The predictive distribution of model performance in a new population can thus be used to characterize uncertainties. If the exchangability assumption does not hold, different steps of this algorithm can be modified to model population differences. If the outcome prevalences are expected to be different; one can shift the distribution of calibrated risks (in Step 4a of Table 1) to match the prevalence in the target population (which itself should be a random variable indicating our uncertainty about prevalence). Independently, if evidence is extracted from a model development study, and there are concerns about the model being overfitted, one can add a negative penalty term to the calibration slope (in Step 4b of Table 1) representing our knowledge about the degree of overfitting. In situations where empirical evidence on the values of such modifying parameters is not available, structured and systematic elicitation of expert opinion is warranted, and typically, dedicated sensitivity analyses are required to examine a plausible range of such parameters [25]. Such a process can benefit from a structured examination of the degree of relatedness between the target population and source population(s), which can help the experts decide on the need for, and extent of, modifications [26].

#### 2.4.2 Sampling from Pre‐Posterior Distributions

In our mixed Bayesian‐likelihood (hybrid) approach, reflecting current practices, we assume that once the future validation sample is obtained, the investigator will solely rely on it for model performance assessment. As such, in each Monte Carlo iteration one instance of $D_{N}$ is created, from which performance metrics are estimated. These are then processed according to the sample size rules of interest (e.g., average CI widths for the expected CI width criteria, the corresponding quantile for assurance‐based criteria, or VoI for NB—details are provided in the next section).

In addition to this default approach based on simulating $D_{N}$, we propose an asymptotic two‐step approach for CI width targets that does not involve simulating individual‐level data. Let $\theta_{k}$ be the metric of interest (e.g., c‐statistic) among θs. Let ${\hat{\theta }}_{k}$ be its sample estimates from the anticipated $D_{N}$. The Central Limit Theorem indicates that

$${\hat{\theta }}_{k}∼\text{Normal}(\theta_{k},SE(\theta_{k})),$$

asymptotically, where $SE(\theta_{k})$ is the relevant SE equation (see Section 2.3). Therefore, one can directly draw from the distribution of ${\hat{\theta }}_{k}$ without simulating $D_{N}$. Within an iteration of the Monte Carlo algorithm, after drawing a value for θ, we obtain a draw from the above distribution as a realization of ${\hat{\theta }}_{k}$ (without simulating $D_{N}$), which is plugged into the SE equation again to quantify its CI width. This approach avoids simulation individual‐level data by using the SE equation twice, which can potentially offer a computational advantage, especially for large sample sizes.

For calculations related to NB, given that sample estimates of prevalence, sensitivity, and specificity all depend on the four frequencies $\left\{N_{tp},N_{fn},N_{tn},N_{fp}\right\}$ (the number of true positives, false negatives, true negatives, and false positives, respectively), $D_{N}$ can be fully specified by these four frequencies, which in turn can be drawn from their respective binomial distributions without simulating individual‐level data.

#### 2.4.3 Sample Size Rules

Because the Bayesian approach takes estimates of model performance in the future validation study as random quantities, it invites establishing sample size rules that take into account such randomness. For metrics related to discrimination and calibration, we consider two sets of rules: Those that target expected precision intervals, and those that target a probability (assurance) that the anticipated precision will be at least as good as targeted. For NB as a measure of clinical utility, we suggest sample size rules that are not precision‐based and rather hone in on our ability to detect the strategy with the highest NB.

##### 2.4.3.1 Sample Size Rules for Metrics of Discrimination and Calibration

Broadly speaking, we consider some scalar summary derived by applying a summarizing function g(.) to validation data $D_{N}$, to meet some criterion. In our context, $g(D_{N})$ returns the 95% CI width for the corresponding metric.

*Expected CI widths (ECIW)*: This is related to the Average Length Criterion discussed by Joseph et al. [14]. Here, we target the expected CI width across the distribution of $D_{N}$:

$$E_{CIW}(N)={𝔼}_{\theta }[{𝔼}_{D_{N}|\theta }(g(D_{N}))].$$

Given that in our Monte Carlo simulation approach, we create S copies of $D_{N}$, this expectation can directly be based on averaging the CI width for each copy of $D_{N}$ created across Monte Carlo simulations. If the sample size is fixed, $E_{CIW}(N)$ is reported for each component as the expected precision of the future study. For sample size calculation, we seek the minimum N that results in $E_{CIW}(N)$ meeting precision target τ: $\min\{N\in \mathbb{N}|E_{CIW}(N)\leq \tau \}$. We derive Ns separately for each component, and choose the maximum N as the final sample size. For example, we might identify the N required to ensure that the expected CI widths of the calibration slope and c‐statistic both meet a desired expected CI width.

*Assurance‐type rules based on quantiles of CI width (QCIW)*: This is related to the modified Worst Outcome Criterion as discussed by Joseph et al. [14]. Here, we target the probability of meeting (or exceeding) desired precision targets. For a given sample size, this approach returns the CI widths corresponding to a desired quantile q (e.g., q=0.9 for 90% assurance):

$$Q_{CIW}(N,q)=F_{N}^{-1}(q),$$

with

$$F_{N}(x)=P(g(D_{N})\leq x)={𝔼}_{\theta }[{𝔼}_{D_{N}|\theta }(I(g(D_{N})\leq x))],$$

being the CDF of the distribution of the anticipated CI width for sample size N. Again, for a fixed‐N setup we report $Q_{CIW}(N)$. For sample size calculation, we find the minimum N such the $q^{th}$ quantile is not greater than the target CI width τ: $\min\{N\in \mathbb{N}|Q_{CIW}(N,q)\leq \tau \}$.

##### 2.4.3.2 Sample Size Rules for NB

*Optimality Assurance for NB*: This is the probability that we will *correctly* identify the strategy that has the highest population NB based on $D_{N}$. To proceed, let $NB_{D_{N}}(k)$ be the sample estimate of NB(k), and $NB_{\theta }(k)$ its true value given θ. We note that with the future data at hand, the investigator will declare a winning strategy as the one that has the highest expected NB solely based on the sample:

$$W(D_{N})=\arg\underset{k}{\max}(NB_{D_{N}}(k)).$$

Optimality Assurance for NB ($A_{NB}$) is the probability that the NB of this strategy is the maximum possible NB:

$$\begin{array}{ll} A_{NB}(N) & =P\left(NB_{\theta }(W(D_{N}))=\underset{k}{\max}(NB_{\theta }(k))\right)\\ & ={𝔼}_{\theta }\left[{𝔼}_{D_{N}|\theta }I\left(NB_{\theta }(W(D_{N}))=\underset{k}{\max}(NB_{\theta }(k))\right)\right]. \end{array}$$

This assurance is non‐decreasing and asymptotes to 1 on the probability scale as the sample size is increased.

*Expected Value of Sample Information (EVSI)*: The key VoI quantity is EVSI(N), the expected gain in NB from conducting a future validation study of size N, compared with the NB of the decision made with current information: 


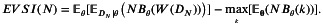


The first term on the right‐hand side is the expected NB of the decision that we will declare as optimal based on $D_{N}$. The second term on the right‐hand side is the expected NB of the best decision under current information ‐ taken to be the decision with the highest expected NB given current information. However, it might be the case that without the validation study, the model will not be implemented, regardless of its potential superiority under current information. In which case, this term can be replaced by the NB of the default strategy (e.g., treating no one or treating all).

EVSI(N) is a non‐decreasing function. Its maximum value occurs when N is infinity; that is, we have access to the entire population and can unequivocally determine the optimal strategy. The expected NB gain from such perfect information is called the Expected Value of Perfect Information (EVPI) and can be calculated as [27, 28]:

$$EVPI={𝔼}_{\theta }[\underset{k}{\max}(NB_{\theta }(k))]-\underset{k}{\max}[{𝔼}_{\theta }(NB_{\theta }(k))].$$

EVSI and EVPI are in the same units as NB (true or false positive). As these units are context‐specific, it is more natural to propose a unit‐less metric as a target of the sample size rule. We propose “relative” EVSI (rEVSI) as the ratio of EVSI to EVPI. This value is intuitive and can be presented as a percentage. An rEVSI of 0.8 for a given sample size means that the external validation study at this sample size is expected to reduce the expected NB loss due to uncertainty by 80%.

For the VoI analysis, we are not aware of any previous work proposing Optimality Assurance. As well, the previously proposed algorithms for validation EVSI were fully Bayesian. Our proposed algorithm for computing Optimality Assurance and EVSI for the mixed Bayesian‐likelihood setup is presented in Table 2.

**TABLE 2:** Computation of optimality assurance and EVSI.

1–4 Generate S draws θ: $\theta^{\left(j\right)}=\{\phi^{\left(j\right)},c^{\left(j\right)},h^{\left(j\right)}\},j=1,2,\ldots ,S$ from Steps 1–4 of Table 1.
5. For j = 1 to S (number of Monte Carlo simulations)
(a) Derive the parameters of the distribution of calibrated risks given the draws $\phi^{\left(j\right)}$ and $c^{\left(j\right)}$.
(b) Calculate true sensitivity and specificity as follows^a^:
$\left\{\begin{array}{l} se^{\left(j\right)}=[\int_{h^{\left(j\right)}(z)}^{1}pf^{\left(j\right)}(p)dp]/\phi^{\left(j\right)}\\ sp^{\left(j\right)}=[\int_{0}^{h^{\left(j\right)}(z)}(1-p)f^{\left(j\right)}(p)dp]/(1-\phi^{\left(j\right)}), \end{array}\right.$
where $f^{\left(j\right)}()$ is the PDF of the distribution of calibrated risks, and $h^{\left(j\right)}()$ is the calibration function, in the $j^{th}$ iteration.
(c) Calculate true NBs ($NB^{\left(j\right)}(k);k\in \{0,1,2\}$) from prevalence, sensitivity, and specificity using Equation (2.2). Record maximum expected NB: $NBmax^{\left(j\right)}=\max_{k}NB^{\left(j\right)}(k)$.
(d) Generate ${D_{N}}^{\left(j\right)}$, defined by $\left\{N_{tp}^{\left(j\right)},N_{fn}^{\left(j\right)},N_{tn}^{\left(j\right)},N_{fp}^{\left(j\right)}\right\}$ as:
$N_{+}^{\left(j\right)}∼\text{Binomial}(N,\phi^{\left(j\right)})$ (number of positive cases in the future sample),
$N_{tp}^{\left(j\right)}∼\text{Binomial}(N_{+}^{\left(j\right)},se^{\left(j\right)})$,
$N_{fn}^{\left(j\right)}=N_{+}^{\left(j\right)}-N_{tp}^{\left(j\right)}$,
$N_{tn}^{\left(j\right)}∼\text{Binomial}(N-N_{+}^{\left(j\right)},sp^{\left(j\right)})$,
$N_{fp}^{\left(j\right)}=N-N_{+}^{\left(j\right)}-N_{tn}^{\left(j\right)}$.
(e) Calculate sample esaimtes of NBs: ${NB}_{D_{N}^{\left(j\right)}}(k);k\in \{0,1,2\}$, by plugging in the sample estimates of prevalence, sensitivity, and specificity from ${D_{N}}^{\left(j\right)}$ in Equation 2.2.
(f) Identify the winning strategy in the sample: $W^{\left(j\right)}={\text{argmax}}_{k}{NB}_{D_{N}^{\left(j\right)}}(k)$.
(g) Record the true NB of this strategy $NBsample^{\left(j\right)}={NB}_{\theta }(W^{\left(j\right)})$.
(h) Record whether the winning strategy had the highest possible NB: $A^{\left(j\right)}=I({NB}_{\theta }(W^{\left(j\right)})=NBmax^{\left(j\right)})$.
Next j
6. Compute the proportion of times the winning strategy has the highest true NB: $A_{NB}=\sum_{j=1}^{S}A^{\left(j\right)}⁄S$. This is the Optimality Assurence.
7. Average NBs from Step 2: $ENB(k)=\sum_{j=1}^{S}NB^{\left(j\right)}(k)/S$. Pick the strategy that has the maximum ENB: $maxENB=\max_{k}ENB(k)$. This is the expected NB of the best strategy under current information.
8. Average NBmaxs from Step 2: $ENBmax=\sum_{j=1}^{S}NBmax^{\left(j\right)}/S$. From this subtract maxENB. This is EVPI.
9. Average NBsamples from Step 2: $ENBsample=\sum_{j=1}^{S}NBsample^{\left(j\right)}/S$. From this, subtract maxENB. This is EVSI.

^a^ The integrals for sensitivity and specificity require numerical methods (with some exceptions, e.g., for the Beta distribution).

Another sample size rule can be based on the Expected Net Benefit of Sampling (ENBS) [29]. The ENBS quantifies the population‐level expected gain from a future validation study over the lifetime the model is to be used (e.g., before the next iteration of the guidelines), minus the cost of sampling [30]. Let ℳ be the expected number of patients for whom the prediction model will be used (this quantity is dependent on the target population size, life expectancy of the model, and its adoption rate—see [30]). The population gain from a future validation study is the EVSI (the net proportion of true positives or false positives) multiplied by ℳ. Let 𝒲 transform the efforts for recruiting one more patient in the same unit as NB. Total efforts of conducting a validation study is thus 𝒲N. The ENBS is the net of these two values:

ENBS(N)=ℳEVSI(N)−𝒲N,

and the optimal sample size is the one that maximizes ENBS. This rule for sample size determination is fully decision‐theoretic as it avoids specifying any arbitrary threshold values. However, it requires scaling NBs to the population and establishing the trade‐off between sampling efforts and clinical utility; both of these tasks are context‐specific. As such, while we propose this rule here for completeness, we will not investigate it in the case study.

#### 2.4.4 Implementation

The above algorithms are implemented in the accompanying *bayespmtools* package [31] (https://github.com/resplab/bayespmtools), in particular as two functions *bpm_valprec()* and *bpm_valsamp()*, for, respectively, computing precision/VoI for a fixed sample size, and determining the sample size corresponding to a set of precision/VoI criteria and sample size rules. These functions expect evidence to be parameterized via four probability distributions, one for the outcome prevalence, c‐statistic, calibration slope, and one of calibration intercept, O/E ratio, or mean calibration. Missing correlation among these distributions are optionally (active by default) imputed via the parametric bootstrap method explained earlier. The correlation is induced to marginal draws for these parameters via the methods by Iman and Conover [32]. This method offers the flexibility to use different distribution types for each component (as opposed to a single multivariate distribution). Both algorithms currently implement Logit‐normal, Probit‐normal, or Beta for the distribution of calibrated risks.

For a specified sample size, *bpm_valprec()* computes expected CI widths, quantiles of CI widths, as well as Optimality Assurance and EVSI using the above‐mentioned Monte Carlo sampling algorithms. *bpm_valsamp()* solves for minimum N that satisfies any of the requested precision criteria coupled with a sample size rule (e.g., targeting an expected CI width of 0.2 for O/E ratio, or 90% assurance that c‐statistic CI width <0.1). Because for any N, one iteration of the Monte Carlo simulation generates one draw from the pre‐posterior distribution of CI widths or NBs, finding the minimum N that satisfies a given sample size rule is a stochastic root‐finding problem. In our current implementation, for targets related to CI widths (expected value and assurance), we use the Robbins–Monro algorithm [33]. This was motivated by the simplicity of this algorithm, which enables simultaneous optimization for all width targets. For Optimality Assurance and VoI metrics, the binomial draws can be vectorized across the entire sample of θs. We use the Stochastic‐Simultaneous Optimistic Optimization algorithm by Valko et al. [34] (implemented in the *OOR* Rpackage [35]).

In their current implementation, these algorithms take <5min on a typical CPU for full sample size calculations (precision and assurance targets for all metrics, and optimality assurance and VoI for NB) based on a Monte Carlo simulation size of 10 000. Parallelization and further improvements in efficiency can reduce this time. Computation times are independent of how predictions are calculated, as these algorithms only require the specification of P(π,θ). Supporting Information—Section 2 provides an illustration on using the *bayespmtools* package for the case study (and replicates, within stochastic margins, the results below) and more details on computational performance.

## 3 Case Study: Predicting Deterioration in Hospitalized COVID‐19 Patients

The ISARIC 4C model is a risk prediction model for predicting deterioration (need for ventilatory support or critical care, or death) in patients hospitalized due to COVID‐19 infection [36]. The investigators used electronic hospital records from all nine health regions of the UK to develop and validate this model. The London region was left out for external validation, and the remaining eight regions were used in internal‐external validation, where one at a time, one region was left out; the model was fitted using data from all other regions, and its out‐of‐sample performance was assessed in the left‐out region [37]. Random‐effects meta‐analysis was used to pool out‐of‐sample estimates of discrimination and calibration metrics. Finally, the investigators used the data from all eight regions to fit one final model, and evaluated its performance in the London region.

To use this setup as an informative example, we assume the London region did not participate in this study, but is now interested in conducting a validation study of this model in their region. That is, we ignore the results reported on the performance of the model in the London region, and consider the internal‐external validation results as the information at hand. We assume that the performance of the model in London region will be a random draw from the distribution of performance observed across other regions (i.e., it is exchangeable with other regions).

The total development sample size was 70 349 (after removing those with unknown outcome status), with a total number of events (deterioration) being 30 316, giving rise to an overall outcome prevalence of 0.428. Pooled estimates of the c‐statistic, mean calibration (difference between average observed and predicted risks), and calibration slope were 0.76 (95% CI 0.75–0.77), −0.01 (95% CI −0.12–0.09), and 0.99 (95% CI 0.97–1.02), respectively. The research question now is: What (minimum) sample size is recommended for a new external validation study in the London region?

### 3.1 Application of Riley Approach

As a baseline comparison, we performed sample size calculations for validating this model using the multi‐criteria method proposed by Riley et al. [3]. Riley et al. used the ISARIC 4C as an example, and for consistency, we use the same targets: Confidence interval widths of 0.1 for the c‐statistic, 0.22 for the O/E ratio, and 0.3 for calibration slope. The assumed true values of these metrics were taken as the summary (pooled) estimates from the above‐mentioned meta‐analyses. The results indicate a sample size of 1056 is required, which is dictated by the calibration slope. For other components, sample sizes are as follows: O/E ratio: 425, c‐statistic: 359.

### 3.2 Application of Bayesian Approach

*Specifying* P(θ): Given our assumption about the exchangeability of populations across UK geographic regions, the predictive distribution of the model performance in a new region represents our uncertainty about the model performance in the London region. However, it is crucial to note that pooled estimates and confidence bands reported in the original study are for the average effect and do not represent predictive distributions. To obtain predictive distributions, we re‐performed the meta‐analyses pooling c‐statistic, mean calibration (note that while we specify evidence in terms of mean calibration, as is reported by Gupta et al., we target O/E ratio for sample size determination), and calibration slope for the eight regions in the internal‐external validation. Gupta et al. did not perform a meta‐analysis on prevalence. We therefore performed this additional meta‐analysis using region‐specific prevalence data from their report. Given the large sample sizes, we performed all meta‐analyses based on the normality assumption on the original scale of each parameter, aside from the c‐statistic, for which a logit‐transformation was performed as recommended by Snell et al. [38] On the other hand, we assigned rang‐epreserving types to the predictive distribution: Beta for prevalence, Logit‐normal for c‐statistic, and Normal for mean calibration and calibration slope. We used the method of moments to derive distribution parameters from the mean and SD of the predictive distribution for a new population. This resulted in the distributions for the four parameters presented in Table 3.

**TABLE 3:** Evidence synthesis for the ISARIC study, based on predictive distribution of the internal–external validation results^a^.

Parameter	Distribution	Mean(SD)	95% Credible Interval^b^
Prevalence	Beta (119.64,159.91)	0.428 (0.03)	0.371–0.486
c‐statistic	Logit‐normal (1.1565,0.0412)	0.761 (0.006)	0.746–0.775
Mean calibration	Normal (−0.0093,0.1245)	−0.009 (0.125)	−0.253–0.235
Calibration slope	Normal (0.9950,0.0237)	0.995 (0.024)	0.949–1.042

^a^ We extracted study‐specific estimates from digitized figures from the original report, which enables us to use three significant digits.

^b^ Matched to the 95% prediction interval from the random‐effects meta‐analysis—see text.

We did not model between‐study correlations (e.g., via a joint meta‐analysis of parameters), after an exploratory analysis (examining Spearman's rank correlation coefficient) did not support the presence of strong between‐study correlation.

*Target precision/VoI criteria and sample size rules*: For these metrics, we apply both the expected value and assurance rules. That is, we determine sample sizes that correspond to the expected CI width being equal to our targets (this can be interpreted as a direct Bayesian counterpart of conventional Riley et al's targets). We also demand a 90% assurance to meet or exceed the CI width criteria, representing our preference for obtaining a narrower over a wider CI width. As for clinical utility, we demand a 90% Optimality Assurance at the 0.2 threshold; that is, we desire to be 90% confident that the strategy that will emerge as the best in the validation sample is actually the best strategy in the target population. Finally, we investigate the expected gain in NB for each component of the sample size on the EVSI curve. This is an example of a hybrid use of this framework: CI widths and Optimality Assurance are used as sample size rules, and resulting sample sizes are assessed in terms of their EVSI values.

*Analysis setup*: All analyses were performed in R [39]. We used the *meta* package for random‐effects meta‐analysis of internal–external validation results of ISARIC [40]. Sample size computations were done using the *bayespmtools* package, with 10 000 draws from P(θ), and assuming Logit‐normal distribution for calibrated risks. We used the sample‐based approach for this analysis. Results of the two‐step approach are provided in Supporting Information—Section 3.

*Results*: Table 4 provides the sample size for each component. The expected CI width components are close to their frequentist counterparts. The assurance‐based targets result in slightly higher sample sizes, as expected. The final sample size of 1181 is dictated by the assurance component of the calibration slope.

**TABLE 4:** Sample size components based on conventional Riley approach (top row) and the Bayesian approach (bottom rows).

Approach	c‐statistic	O/E ratio	calibration slope	NB
Conventional Riley approach (expected CI widths)	359	425	1056	149
Bayesian (expected CI width)	351	430	1064	NA^a^
Bayesian (90% assurance)	399	522	1181	306

^a^ Not computed as this framework emphasizes computing assurance and VoI metrics for NB.

**FIGURE 1:**
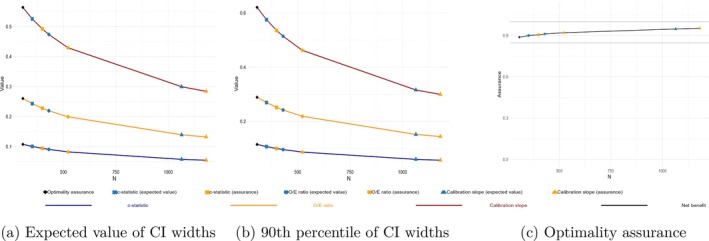
Expected CI widths (a), 90th quantile of CI width (b), and optimality assurance (c) curves. Shapes on the line pertain to individual sample size components. (a) Expected value of CI widths, (b) 90th percentile of CI widths, and (c) Optimality assurance.

Figure 1 shows how the precision targets change with sample size. These are computed from a separate call to *bayespm_prec()* (independently of the calculations that determined the sample sizes) and, as such, act as diagnostic plots.

**FIGURE 2:**
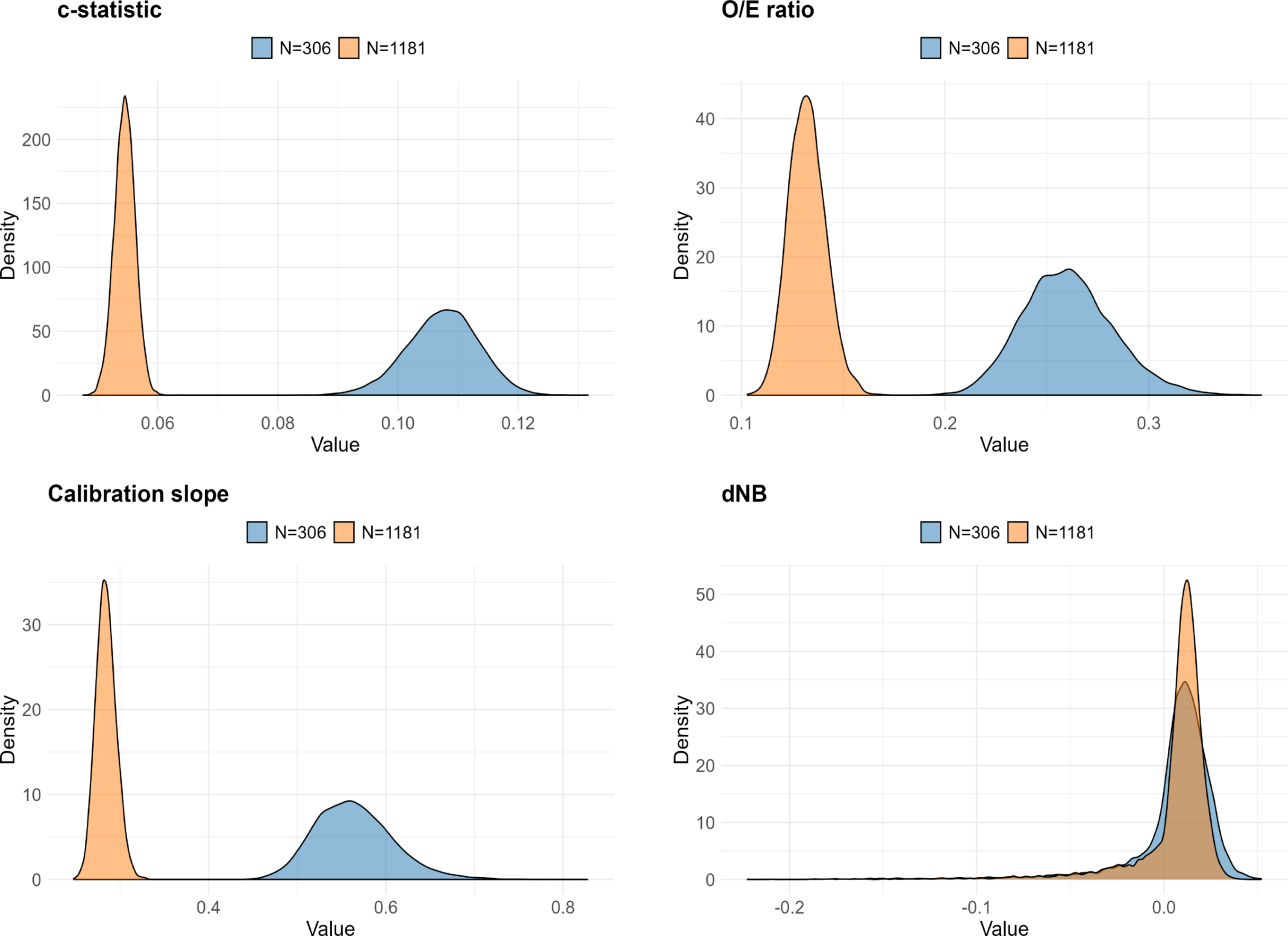
Kernel histograms of CI widths for (a) c‐statistic, (b) O/E ratio, and (c) calibration slope. Also, (d) shows the kernel histogram of the incremental net benefit of the model compared to the best default strategy for two sample sizes.

Figure 2 shows the exemplary kernel histograms of the distribution of CI widths (the first three panels) for the smallest (N=306) and largest (N=1181) components of the sample size. The last panel demonstrates the distribution of the incremental NB of the model compared with the default strategies (i.e., $NB_{1}-\max(NB_{0}-NB_{2})$). With higher sample sizes, the CI widths get both shorter and more clustered. The distributions are relatively symmetrical. This can explain why the ECIW values are close to their conventional, frequentist counterparts. For NB, as the sample estimate of NB is an unbiased estimator, higher sample sizes will result in a narrower distribution, but their location remains the same. For Optimality Assurance and EVSI, what affects the results is the share of the distribution that falls on the left side of zero.

**FIGURE 3:**
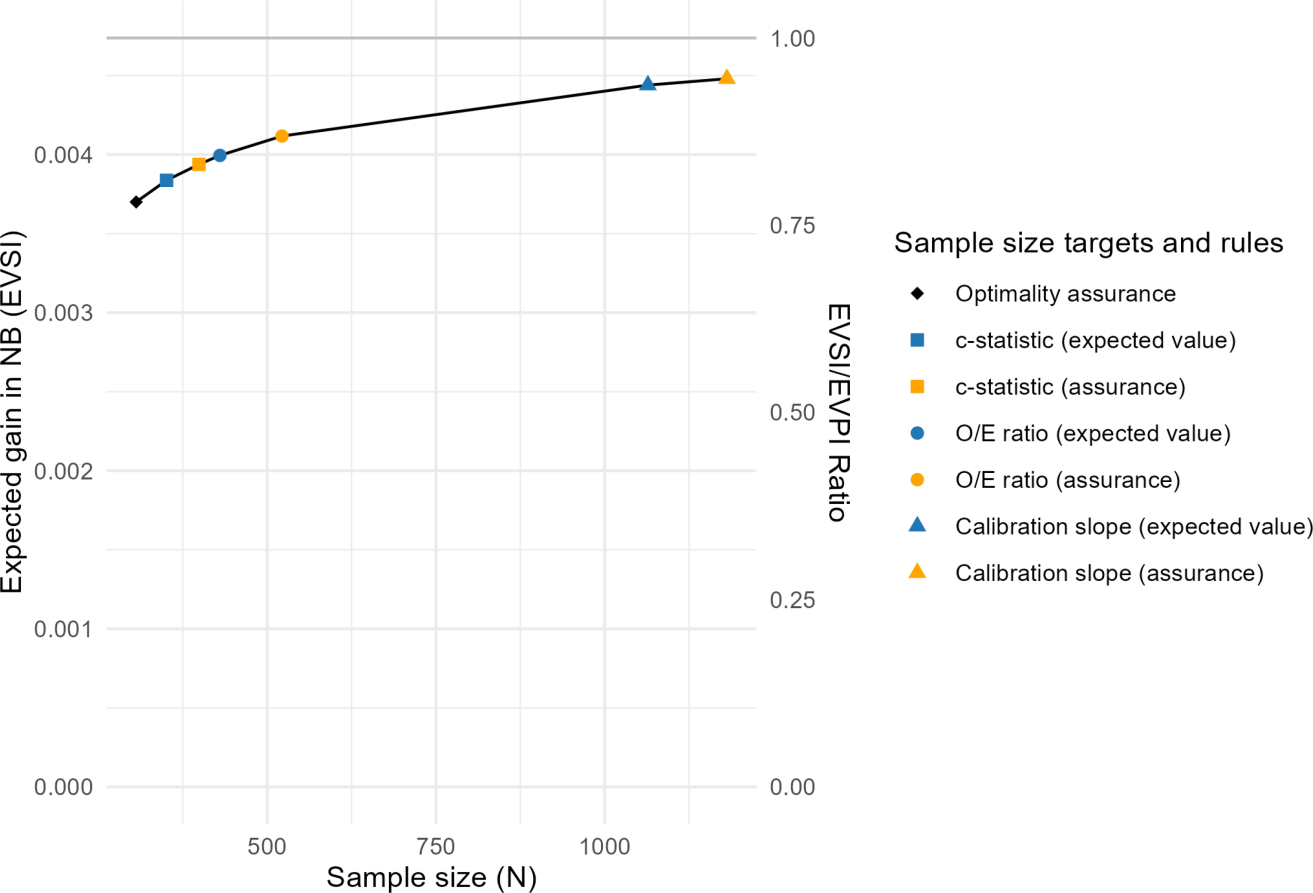
Expected Value of Sample Information (EVSI) curve. Shapes pertain to individual sample size components.

Turning to VoI analysis, Figure 3 provides the EVSI calculation (with EVSI/EVPI ratio [rEVSI] as the secondary Y‐axis). Components of sample size calculation are overlaid on the graph.

From this figure, one can (subjectively) realize that some of the CIW components fall on the “diminishing return” of the EVSI curve. For example, going from the largest sample size based on criteria other than the calibration slope (N=522) to the sample size dictated by assurance on calibration (N=1181), a more than doubling of the sample size, is associated with only 8.8% expected gain in NB. This can justify relaxing the criteria on the calibration slope for the validation study, if the focus is on clinical utility.

**FIGURE 4:**
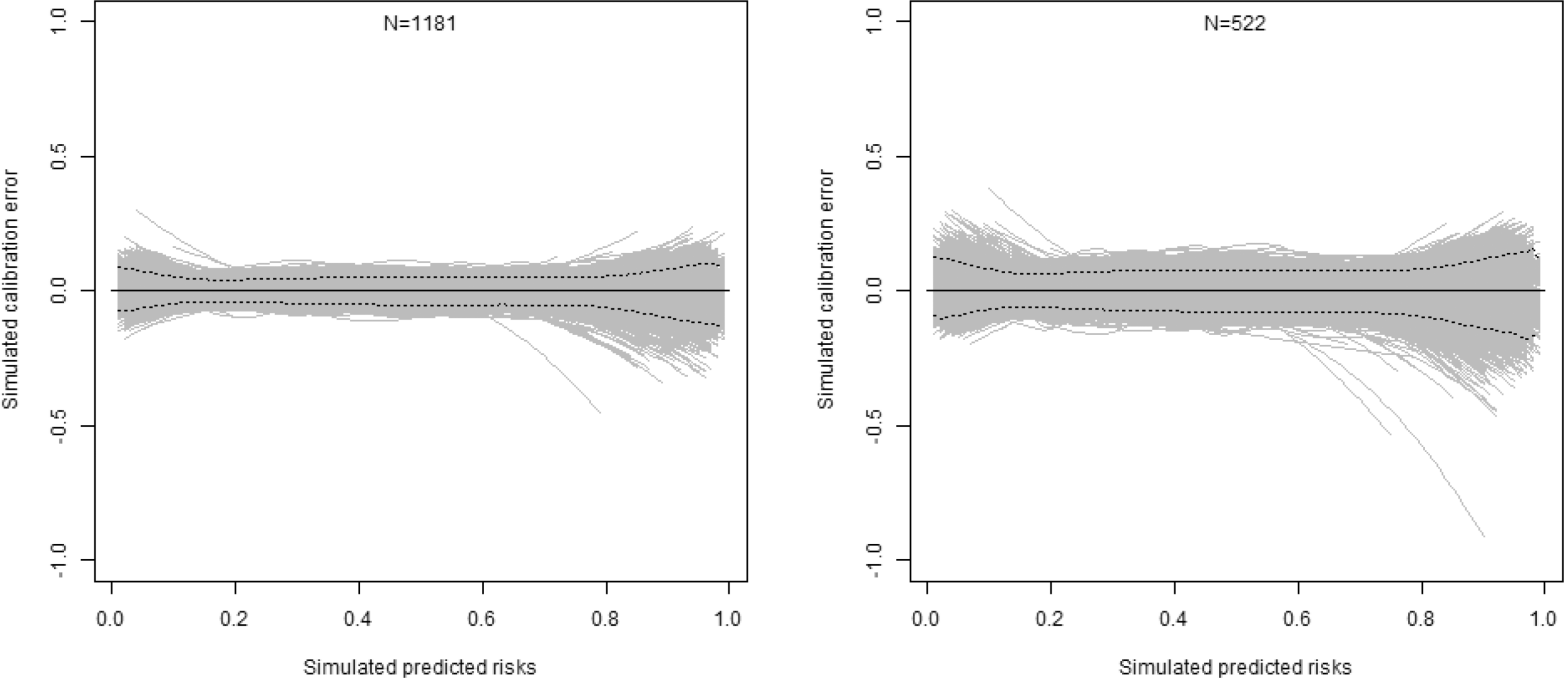
Bayesian calibration error plots—dashed lines are 95% credible bands.

The decision whether to relax the calibration slope criteria can also be examined via the stability of the flexible calibration plots. As the desired precision based on the calibration slope is difficult to justify, but often dictates the final sample size required, Riley et al. recommend plotting the corresponding variability in calibration curves that might arise for that sample size [9]. In our context, the variability of a calibration curve has two sources: The variability in the true calibration function (due to variability in θ) and the variability due to the finite validation sample (due to variability of $D_{N}$). The former is not a function of sample size. Because in our Bayesian Monte Carlo sampling algorithm, the true calibration function is known within each iteration, we can remove variability due to θ to quantify the difference between the flexible calibration curve and the true calibration function: For each simulated $D_{N}$, we fit a kernel regression (e.g., LOESS) for P(Y=1|π), then remove h(π) from the fitted values. Figure 4 shows such Bayesian calibration error plots for the final sample sizes, including calibration slope criteria (N=1181) and after removing such criteria (N=522). Moving to the smaller sample size expectedly increases the spread of calibration errors. Nevertheless, aside from extreme values of predicted risk, the 95% credible intervals (dashed lines) indicate that the error will mostly be <0.1. Again, this can be taken as a further argument in favorof settling on the smaller sample size. Putting all these together, the final recommended sample size will be N=522.

## 4 Discussion

We proposed a Bayesian framework for sample size considerations when a risk prediction model is to be evaluated (validated) in a target population. Compared to the conventional frequentist framework, this approach offers several advantages: It allows investigators to specify their uncertainties about model performance, in addition to the sample size rules targeting expected CI widths, it enables assurance‐type sample size rules that incorporate the investigators' risk preferences, and it facilitates the use of Value of Information analysis when clinical utility of the model is being examined. We proposed characterizing uncertainties around common metrics of model performance, and introduced Monte Carlo sampling algorithms for computing precision and VoI metrics. We implemented this approach as the *bayespmtools* R package, with two functions, one for quantifying the anticipated precision/VoI values given a fixed sample size, and one for determining the sample size given pre‐specified rules.

Overall, this framework can provide a transparent, reproducible, and justifiable approach when designing validation studies aimed at quantifying the performance of a pre‐specified model in terms of trade‐off between sample size, precision, and gain in clinical utility. This framework can be used to investigate the anticipated precision or expected gain in clinical utility when the sample size is fixed (based on already collected data). It can also be used to determine the sample size when the original data collection is planned. A hybrid use, determining the sample size based on certain criteria, and examining the resulting precision and VoI for other outcomes is also possible. Our case study demonstrated such a hybrid approach. We defined rules targeting the expected value and 90% assurance on the widths of confidence intervals around metrics of discrimination and calibration, as well as 90% assurance that we will be able to correctly identify the strategy with the highest clinical utility. Then, based on the results of the VoI analysis (as well as visual inspection of calibration errors), we could argue that one could potentially relax one component of the sample size requirement (precision around calibration slope), without losing much precision or clinical utility.

Among the outputs of this Bayesian approach, the assurance‐type rules and VoI metrics are particularly relevant for risk prediction modeling. If the estimates of model performance are too uncertain, the adoption of the model might be met with resistance, even if the point estimates are compatible with acceptable performance. This, combined with the general risk‐averse attitude of public funding and decision‐making agencies [41], means the costs of not meeting the desired targets are typically more than the benefits of exceeding those targets. Assurance‐type rules can be used alongside those based on expected values to assuage concerns that the planned study might generate ambiguous results. Further, VoI metrics address longstanding criticisms on the irrelevance of inferential statistics when deciding on whether to adopt a health technology (including markers and risk scoring tools) for patient care [10]. These criticisms have been recently voiced in the particular case of NB calculations for risk prediction models [11]. Accordingly, we recommend sample size considerations around NB to move away from confidence intervals and focus on Optimality Assurance and VoI analysis. In particular, EVSI combines the risk of failing to identify the optimal strategy with the consequences (NB losses) of such a failure, providing a fuller picture of the implications of learning from an empirical study in terms of clinical utility. This provides a “value‐based” perspective that can complement the precision‐based approach for sample size determination around metrics of discrimination and calibration.

There are several areas for further inquiry. We focused on binary outcomes, but this methodology can be extended to other outcome types. The application to survival outcomes seems particularly relevant and feasible. For such outcomes, model performance needs to be assessed at a time point of interest [42]. The time‐dependent equivalents of prevalence and c‐statistic are, respectively, the complement of survival probability and the time‐dependent area under the Receiver Operating Characteristic curve [43]. If uncertainty around these metrics is specified, our identifiability conditions can then be employed to map such metrics to the distribution of calibrated risks at this time point. However, the impact of censored observations in the future sample and the presence of competing risks on precision metrics and VoI values remains to be investigated. We focused on precision and VoI targets that pertain to the population‐level performance of the model. Sample size consideration can also be approached in terms of uncertainty at the individual level, such as instability at individual‐level predictions [44]. Further, when contemplating a validation study, often a secondary objective is to update (revise) the model if its performance turns out to be sub‐optimal. It makes sense to consider targets related to model updating when designing validation studies [45]. In line with the Riley equations, we modeled the calibration function to be linear on the logit scale. This can be relaxed, for example, by introducing a quadratic term as long as one can assure monotonicity of h() and specify the joint distribution of the three parameters that would define it. A more appealing extension would be to model h() non‐parametrically. This can be done based a non‐parametric summaries of calibration curves, such as those based on kernel smoothing (e.g., ICI[20]) or cumulative plots (e.g., $C^{*}$[46]).

From an implementation perspective, Bayesian calculations are inherently more complex than their frequentist counterparts. Several components of this framework require numerical integration and optimization methods. Examples include finding the parameters of P(p) given prevalence and c‐statistic, mapping O/E ratio or calibration mean to calibration intercept, and computing sensitivity and specificity (for NB calculations) at the threshold of interest given P(p) and h(). These numerical algorithms might struggle with extreme cases. In addition, as the Bayesian Monte Carlo sampling produces draws from the pre‐posterior distributions of precision targets (e.g., CI widths), sample size determination requires stochastic optimization techniques. These algorithms demand convergence assessment and, if needed, repetition with different starting values. While our implementation performed robustly in our examinations (including the case study), numerical accuracy and stability of these algorithms should be considered across a range of input values in dedicated studies. We consider the accompanying software to be an evolving implementation, subject to revisions and improvements.

Compared to the conventional sample size determination methods, the Bayesian approach is predicated on one crucial extra step: Characterizing our uncertainties about model performance. Such characterization is not currently a common practice in applied prediction modeling. The conventional wisdom in risk modeling studies is to report a “final model” as a deterministic function that maps patient characteristics to an exact value of conditional outcome risk. This practice ignores the fact that predictions for a model trained using a finite sample are inherently uncertain. Differences across populations in the relationship between predictors and the outcome, differences in predictor assessment approaches across settings, and variations in outcome ascertainment methods further add to our uncertainties [47]. Fortunately, there have been recent calls on the importance of characterizing and communicating uncertainty around model predictions [48]. These calls encourage investigators to fully document and communicate uncertainty in model coefficients in reports of model development (e.g., reporting the covariance matrix of model coefficients, or reporting the coefficients of models fitted in bootstrapped copies of the original sample) [48]. These can further facilitate incorporating uncertainties into the design of subsequent studies.

A thought‐provoking consequence of adopting a Bayesian approach is the ultimate fate of the prior information. Reflecting current practices, we assumed the future validation sample will be the sole source of evidence on model performance once it is procured, in effect discarding our current knowledge once it is used to construct P(θ). But, if our existing knowledge is informative enough to warrant investigating the model in a new setting, should it not be used alongside the sample to inform our final judgment? Taking our case study as an example, the information on prevalence alone (based on the moments of its distribution) is equal to having learned its value from a sample of 281 individuals—a non‐trivial amount of information! Incorporating such prior knowledge into what we learn from the data will reduce prediction uncertainty, which can affect both patient care (more precise predictions) and the design of empirical studies (requiring smaller samples to achieve the same targets). Of course, a perceived drawback is the subjectivity inherent in real‐world Bayesian reasoning. Our case study might be seen as a stylized setup where predictive distributions from a meta‐analysis were available, and the assumption of exchangeability of populations seemed defendable. Things can be more subjective, for example, when a single development study in another population is the sole source of evidence. Our current response to the fear of compromised objectivity is to completely drop the existing information. Perhaps one can instead define objectivity in terms of explicit and transparent specification of prior evidence and steps taken to account for population heterogeneity.

Overall, a cultural shift towards embracing uncertainty in predictions may also encourage the adoption of study designs that more effectively leverage existing knowledge. We expect Bayesian approaches towards the study design to gain traction as awareness grows regarding the relevance of prediction uncertainty in decision making.

## Funding

This work was supported by the Canadian Institutes of Health Research (Grant No. PHT 178432), Natural Sciences and Engineering Research Council of Canada (Grant No. RGPIN‐2019‐03957), Talent Programme financed by the Dutch Research (Grant No. VIDI 9150172310023), UK Engineering and Physical Sciences Research Council (Grant No. EP/Y018516/1), and UK Medical Research Council ‐ National Institute for Health and Care Research Better Methods Better Research (Grant No. MR/Z503873/1).

## Conflicts of Interest

The authors declare no conflicts of interest.

## Supporting information




**Data S1.** Supporting Information.

## Data Availability

The data that support the findings of this study are openly available in GitHub at: https://github.com/resplab/papercode/tree/main/BayesSS.

## Appendix A Details of Net Benefit (NB) Calculations

To turn a continuous predicted risk into a binary classification to inform a treatment decision, a decision maker needs to specify a risk threshold z on predicted risks, such that individuals classified as high risk are selected for the treatment (e.g., a 10% threshold (z=0.1) on The predicted 10‐year risk of cardiovascular diseases is often used to initiate treatment with statins to reduce cardiovascular disease risk).

Consider a cohort of patients whose outcome risk is precisely z. For this cohort, a decision maker would be ambivalent between treatment and no treatment. Considering this cohort as test positive and treating them would mean correctly treating the proportion z who will experience the event (i.e., true positives) but unnecessarily treating the remaining 1−z proportion (i.e., false positives). The ambivalence between not treating this cohort implies that the decision‐maker considers the utility of the two decisions to be the same. Anchoring the utility of not treating anyone at 0, this ambivalence therefore means the decision‐maker assigns a relative utility weight of −z/(1−z) to each false positive classification compared with each true positive classification. Consequently, the NB of the model at threshold z, compared with treating no one, can be calculated in net true‐positive units as:

$$\begin{array}{ll} NB & =P(\text{True Positive})-\frac{z}{1-z}P(\text{False Positive})\\ & \;=se\phi -\frac{z}{1-z}(1-\phi )(1-sp). \end{array}$$

In addition to not treating anyone or using the model, there is a third option of treating all, whose NB can be calculated by conceptualizing it as using a test with sensitivity of 1 and specificity of 0:

$$NB=\phi -(1-\phi )\frac{z}{1-z},$$

thus giving rise to the NB equation in the main text (2.2).
