# Supplementary material for: Bayesian Sample Size Calculations for External Validation Studies of Risk Prediction Models
Source: Stat Med. 2026 Feb 11;45(3-5):e70389. doi: 10.1002/sim.70389 (PMC12894519; doi:10.1002/sim.70389)
Supplement: Supplementary file 1 — Data S1. Supporting Information. [file SIM-45-0-s001.pdf]

Supplementary Material for 'Bayesian sample size calculations for external validation studies of risk prediction models'

January 01, 2026

Table of Contents

Section 1: Algorithm for parametric bootstrapping

Section 2: Ilustrative example of using bayespmtools and computational benchmarks

Section 3: Sample size and CI widths for the two-step approach

Section 4: Code and data availability

Section 1: Algorithm for parametric bootstrapping

The algorithm below creates, repeatedly, simulated validation samples of size n based on the point estimates of prevalence, discrimination, and calibration from the original study. Generally, correlation coefficients are not sensitive to the size of the sample. It is natural to choose n to be the size of the original sample. In instances (as in our case study) where elements of θ are taken from different sources or from the output of meta-analysis (and therefore n is not fixed), one can determine n based on the effective sample size, for example based on uncertainty around prevalence. This results in $n = 280$ for the case study, given the SE around prevalence.

1. For i in 1 to M (size of the Monte Carlo simulation)
   (a) Generate $D_n := \{(\pi_i, Y_i)\}_{i=1}^{n}$, an iid sample of size $n^{\dagger}$ consisting of predicted risks and response values using the point estimates $\hat{\theta} = \{\hat{\phi}, \hat{c}, \hat{h}\}$.
   (b) Estimate $\theta^{(j)} = \{\phi^{(j)}, c^{(j)}, h^{(j)}\}$ from the above sample.
2. Estimate the correlation coefficients for θs.

Section 2: Ilustrative example of using *bayespmtools* and computational resource use assessment

Note: minor discrepancies with the main results are due to rounding of input parameters and Monte Carlo variability. Sample size calculations for the Riley's approach in the main text are derived using *pmvalsampsize.*

Also, please refer to the tutorials for this package as alternative (and p)

```
set.seed(123)

#Evidence from the ISARIC study
#See the main text (and analysis code) for how these distributions are derived.
evidence <- list(
  prev~beta(mean=0.427966984132821, sd=0.0295397309129426),
  cstat~logitnorm (mean=0.760628336908955, sd=0.00635806041351944),
  cal_mean~norm(mean=-0.00934717199436785, sd=0.124517605045825),
  cal_slp~norm(mean=0.995017759715243, sd = 0.0237278675967507))


#Specifying targets
#eciw=x indicates desired expected CI Width of x.
#qciw=c(a,b) indicates desired assurance CI Width of x at assurance level y.
targets <- list(eciw.cstat=0.1,
                eciw.cal_oe=0.22,
                eciw.cal_slp=0.30,
                qciw.cstat=c(0.9, 0.1),
                qciw.cal_oe=c(0.9, 0.22),
                qciw.cal_slp=c(0.9, 0.3),
                oa.nb=0.9)

library(bayespmtools)

#Main function call
samp <- bpm_valsamp(evidence=evidence,      #Evidence as a list
                    dist_type="logitnorm",  #Distribution type for calibrated risks
                    method="sample",        #Sample based or tw-level ("2s") method
                    targets=targets,        #Targets (as specified above)
                    n_sim=10000,            #Number of Monte Carlo simulations
                    threshold=0.2)          #Risk threshold for NB VoI calculations


print(samp$results)
```

```
##   eciw.cstat  eciw.cal_oe eciw.cal_slp   qciw.cstat  qciw.cal_oe qciw.cal_slp
##          351          430         1064          399          522         1181
##        oa.nb
##          306
```

Computational resource use

Benchmarking is performed on a Personal Computer with Windows 11 Professional, Intel(R) Core(TM) Ultra 9 285K (3.70 GHz), and 64GB of RAM. Results are average of 10 independent simulations (single-threaded)

for each function call. *bpm_valsamp()* is called with the arguments as in the example above. *bpm_valprec()* is called with a sample size of 1,000, and with other arguments the same as those of *bpm_valsamp()*.

Method	Time (s)	Peak memory use (MiB)
valsamp	177.2	84.0
valsamp (two-step)	156.2	84.1
valprec	23.2	84.1
valprec (two-step)	28.6	32.1

Section 3: Sample size and CI widths for the two-step approach

Table below shows the final sample sizes for the same setup for the ISARIC study, but with using two-stage method instead of the sample-based approach.

Approach	c-statistic	O/E ratio	calibration slope
Bayesian (expected CI width)	351	430	1064
Bayesian (90% assurance)	399	522	1181

The Figure below shows the precision, computed independently, for the derived sample sizes (VoI results are not reported as the two-step approach is not applicable to them).

Figure 1: *
Expected CI widths (a) 90th quantile of CI width (b), optimality assurance. Shapes on the line pertain to individual sample size components.

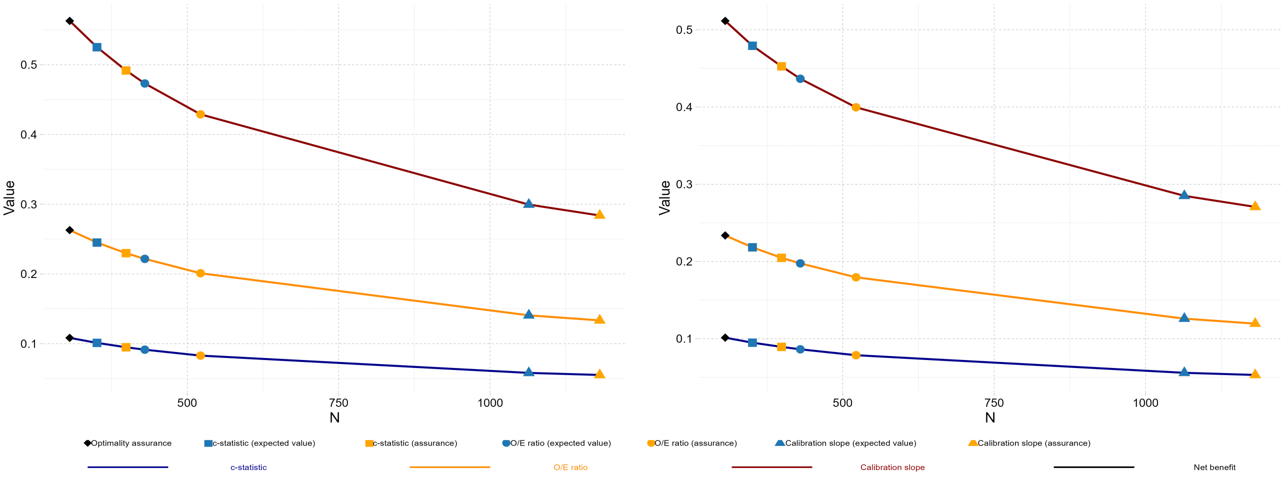

(a) Expected value of CI width

(b) 90th percentile of CI widths

Section 4: Code and data availability

- The core functions are under the *bayespmtools* R package (https://github.com/resplab/bayespmtools).
  - Please install the development version of the package (e.g. via `remotes::install_github("resplab/bayespmtools")`).
  - Note that this package also has dependencies: *fastLogisticRegressionWrap, logitnorm, mc2d, mcmapper, pROC, cobs*
- The code generating the results in the paper is available from `https://github.com/resplab/papercode/tree/main/BayesSS`.
  - The main file is `Main.Rmd`.
  - The code also depends on other packages (eg *ggplot2*) for processing the results.
  - Please make sure `res_path` folder in the first chunk points to a folder on your local system.
